# Effect of Zinc Oxide Modified Silica Particles on the Molecular Dynamics of Carboxylated Acrylonitrile-Butadiene Rubber Composites

**DOI:** 10.3390/polym9120645

**Published:** 2017-11-25

**Authors:** Magdalena Gaca, Joanna Pietrasik, Marian Zaborski, Lidia Okrasa, Gisèle Boiteux, Olivier Gain

**Affiliations:** 1 Institute of Polymer and Dye Technology, Lodz University of Technology, Stefanowskiego 12/16, 90-924 Lodz, Poland; joanna.pietrasik@p.lodz.pl (J.P.); marian.zaborski@p.lodz.pl (M.Z.); 2 Department of Molecular Physics, Lodz University of Technology, Zeromskiego 116, 90-924 Lodz, Poland; lidia.okrasa@p.lodz.pl; 3 Ingénierie des Matériaux Polymères, Universite Claude Bernard Lyon 1, UMR CNRS 5223, 15 Bd A. Latarjet, 69622 Villeurbanne, France; gisele.boiteux@univ-lyon1.fr (G.B.); olivier.gain@univ-lyon1.fr (O.G.)

**Keywords:** carboxylated acrylonitrile-butadiene rubber, molecular dynamics, zinc oxide modified silica, composite, ionic crosslinks

## Abstract

This work examines the molecular dynamics of carboxylated acrylonitrile-butadiene rubber crosslinked with zinc oxide modified silica particles. ZnO/SiO_2_ with the wide range of ZnO concentrations were used as both a crosslinking agent and filler. A series of thermal measurements were applied to the characterization of the samples: differential scanning calorimetry, dynamical mechanical thermal analysis, and dielectric relaxation spectroscopy. A complementary experimental technique, which is equilibrium swelling in solvents, confirms the presence of ionic crosslinks, which are created between zinc ions and the functional carboxyl groups of the rubber, within the structure of the vulcanizates. These interactions influenced not only the affinity of the vulcanizates to solvents, but also their dynamic mechanical and dielectric properties. In these investigations, the influence of concentration of ZnO on the surface of the ZnO/SiO_2_ on the properties of the vulcanizates are described.

## 1. Introduction 

Thermoset elastomers are predominately cured by sulfur vulcanization [1,2,3,4], using sulfur in the presence of activators and accelerators or with organic peroxides [5] to form covalent crosslinks. Such crosslinked elastomers are insoluble composite materials, and contrary to the properties of thermoplastics, these elastomers cannot be reprocessed once they are formed. This provides a motivation to reduce the amount of sulfur curing agent by incorporating ionic crosslinks to generate the elastomeric network. The incorporation of ionic crosslinks provides a beneficial effect on the properties of the elastomers in addition to the fact that they are reversible at higher temperatures [6]. Indeed, the thermolabile nature of ionic clusters leads to their reprocess-ability at higher temperatures, while at room temperature they act as fillers. The ionic interactions generate an unconventional crosslinking reaction, consisting in partial or total neutralization of functional groups incorporated into the rubber [7,8,9,10,11,12,13,14]. For that purpose, one can use metal oxides or other chemical compounds containing substituent groups able to react with macromolecules [14,15,16,17,18]. The crosslinks coming into existence this way have the nature of salts [7,10]. Polymeric materials containing pendant ionic groups are called ionomers [7]. It is possible to form elastomers with different kinds of crosslinks by the selection of the crosslinking agents and crosslinking procedures, which will modify the properties of the ionomers and hence their specific applications [14,19,20]. The exceptional properties of elastomers containing ionic crosslinks have generated extensive interest in the production of such elastomers [6,21,22,23,24,25].

Our research group has been working on the development and evaluation of new materials with a core-shell structure for crosslinking rubbers. [21,22,26] A specific example is carboxylated butadiene-acrylonitrile rubber (XNBR), cured with zinc oxide modified silica particles (ZnO/SiO_2_), which produce ionic bonds as well as covalent ones in the crosslinked vulcanizates.

The main goal of this work was to carry out different thermo-physical measurements to analyze the properties of the XNBR vulcanizates on the molecular level. Differential thermal calorimetry (DSC), dynamic mechanical thermal analysis (DMTA), and dielectric relaxation spectroscopy (DRS) were used for the characterization of the samples. Additional supplementary measurements (equilibrium swelling in toluene and mixture of toluene with ethylenediamine) were also carried out to support the conclusions drawn from thermal data. The effect of both a new crosslinking substance and novel fillers, with a core-shell structure, on the properties of the formed vulcanizates are detailed.

## 2. Materials and Methods

### 2.1. Materials

Details of the materials used in this study are listed in Table 1. Vulcanizates containing XNBR and various crosslinking agents were prepared by crosslinking with dicumyl peroxide (DCP), zinc oxide (ZnO) or with silica particles whose surface was modified with different mole percentages of zinc oxide (ZnO/SiO_2_). 

All of the surface modified silica particles were used as new crosslinking substances and were prepared through wet impregnation of silica with zinc nitrate. Then, after drying, the ZnO/SiO_2_ precursors were calcined in the air at 450 °C during over 4 h [27]. The resulting modified silica particles contained 1, 8, 12, and 20 wt % of zinc oxide on the surface, are named samples 1ZnO/SiO_2_, 8ZnO/SiO_2_, 12ZnO/SiO_2_, and 20ZnO/SiO_2_ respectively. The ZnO content on the surface of the modified silica was determined by inductively coupled plasma using an IRIS-AP spectrometer from Thermo Jarrell Ash (Franklin, MA, USA) with horizontal plasma observation and using a one-component Merck standard.

### 2.2. Preparation of Rubber Blends and Vulcanizates

The compositions of XNBR rubber blends are shown in Table 2. The composition of these blends are provided by specifying the amounts of the compounds as weight % of any additive per hundred parts of rubber (phr). 

Samples were produced using the following protocol: compounds were mixed at ~35 °C using a laboratory two-roll mill with a cylinders diameter of 200 mm and a length of 450 mm. The drilled rolls were cooled by circulating cold water through them during the blending procedure. The rotational speed of the front roll was *V*_p_ = 2 s^−1^ with friction ratio 1.1. First, a gum rubber was masticated for 4 min, and then the ingredients (following compounds in Table 2) were added and mixed continuously. The quality of mixing was maintained by adjusting the gap between the cylinders, time of blending, and uniform cutting operation. After the mixing process, the uncured XNBR blends were sheeted with a thickness of about 6–8 mm and stored at the temperature 2–6 °C for 48 h, and later molded in an electrically heated hydraulic press at 160 °C for half an hour to form the vulcanizates of appropriate thickness. Next, they were stored at room temperature.

### 2.3. Methods of Testing

The crosslinking density (ν) of the vulcanizates was determined from the equilibrium swelling of the vulcanizates immersed in toluene. The crosslinking density, defined as the number of moles of network bonds per volume unit of rubber, was calculated using the Flory-Rehner equation [28]. To determine whether the crosslinked polymers contain a fraction of non-covalent crosslinks, the vulcanized samples were swelled in the mixture of toluene and ethylenediamine at room temperature for a period of 48 h, and then the remaining robust crosslinking density was calculated, (ν_A_) [29]. According to the work of Vondracek et al. [30] and our own observations [21], the presence of amines contributes to the degeneration of the ionic crosslinks formed at the filler-rubber interfaces. The concentration of chemical crosslinks was estimated from the difference between ν and ν_A_. 

The equilibrium swelling data were deduced using the value of *μ*, a parameter of the polymer-solvent interaction, defined as: *μ* = 0.4132 + 0.4341*V*_r_ for XNBR-toluene and *μ* = 0.3537 + 0.5216*V*_r_ for XNBR-toluene + ethylenediamine, where *V*_r_ is the volume fraction of rubber in the swollen vulcanizate. By specifying the *μ* parameter, β and *μ*_0_ values (necessary for calculating the crosslinking density of the chosen samples) are pointed out. In fact, these parameters are determined by the dependence *μ* = *μ*_0_ + β*V*_r_ where β is a straight-line factor describing the relation *μ* = f(*V*_r_) and *μ*_0_ is the parameter of the elastomer-solvent interaction when *V*_r_ = 0.

Differential scanning calorimetric (DSC) measurements were carried out using a Netzsch DSC 200 Instrument (Netzsch, Selb, Germany). Samples weight from 11 to 25 mg were sealed in aluminium pans and then heated with a heating rate of 10 °C/min over the temperature range from −60 to 120 °C, under a nitrogen atmosphere. The glass transition temperatures (*T*_g_) were determined as the midpoint of the step change in the baseline of the recorded DSC signal.

Molecular relaxations were characterized by dynamical mechanical thermal analysis (DMTA), carried out with a dynamic mechanical thermal analyzer type DMA 2980TA Instruments (TA Instruments, New Castle, DE, USA), and by dielectric relaxation spectroscopy (DRS) using DEA 2970 Dupont TA Instruments spectrometer. DMTA was performed according to the film tension mode over the temperature range from −80 to 120 °C, with a temperature ramp of 1 °C/min using rectangular samples with the length up to 18 mm, width ~6 mm, and thickness ~1 mm. The frequencies applied were in range 1–30 Hz. 

The general DRS measurement procedure was to cool the sample to −80 °C, tighten, and hold it for 10 min at this temperature (to reach the equilibrium), and then DRS was performed in the frequency range of 1–50 kHz, and in the same temperature range as DMTA with a programmed heating rate of 2 °C/min. Sputter coated ceramic sensors (electrodes) and circular shape samples with diameter 25 mm and thickness less than 0.8 mm without metallization were used. A 300 N compression force between the ceramic electrodes was applied. Samples were placed in a glass bell jar under nitrogen gas flow to avoid oxidation during the increase of temperature. Analysis of the dielectric results was performed using both, the classical representation of dielectric relaxations, i.e., dielectric complex permittivity ε*, as well as using the electric modulus representation [31]. The real *M′* and imaginary *M″* parts of the electric modulus were calculated according to the Equations (1) and (2):

*M′* = *ε′*/(*ε′2* + *ε″2*) (1)

*M″* = *ε″*/(*ε′2* + *ε″2*) (2)

where: *ε′* and *ε″* are, respectively, the real and imaginary parts of complex permittivity ε*, which fulfill the following equation:

ε* = *ε′ + iε″* (3)

The relaxation maps were prepared on the basis of both dielectric and mechanical spectra. The points were determined manually from position of the maxima of the *M″*(*T*) dependence for DRS results and the *E″*(*T*) dependence from the mechanic results. 

## 3. Results and Discussion 

ZnO modified silica particles (ZnO/SiO_2_) were used, not only as a filler, but also as a crosslinking agent for XNBR. We presented in our previous paper that the amount of ZnO applied to modify the surface of precipitated silica was varied from 1 to 20 wt %, resulting in a variation in the crosslinking density of the vulcanizates (ν) (Table 3) [21]. 

**Scheme 1:**
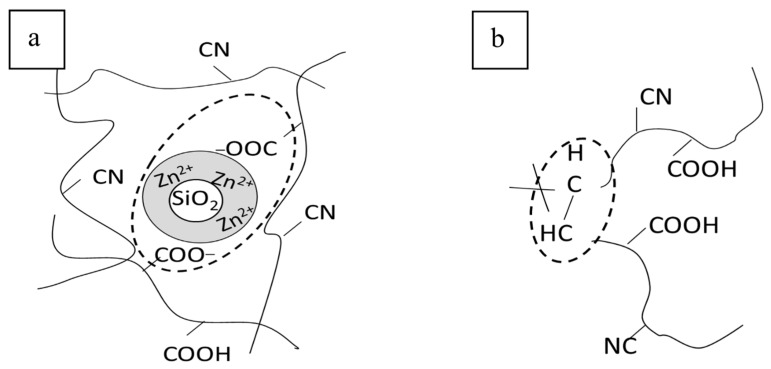
The structure of crosslinks in the XNBR vulcanizates when (**a**) silica modified with zinc oxide and (**b**) dicumyl peroxide were used to crosslink rubber.

Earlier mentioned investigations had confirmed the presence of crosslinks in the resulting elastomer network, which do not have any covalent character (Scheme 1). These ionic interactions were formed between zinc ions and the carboxyl groups of the rubber and were disintegrated to some extent under toluene-ethylenediamine treatment, which led to a decrease in the crosslink density (Table 3) [21]. For comparison, when the sample was crosslinked by dicumyl peroxide, without adding any silica filler, only covalent C–C bonds were created between the rubber chains (see Scheme 1). 

DSC measurements were carried out on the different vulcanizates (except for sample X12mS), and a summary of the results is given in Table 4. It is clear that introducing ZnO/SiO_2_ as crosslinking substance into the compounded rubber led to no significant changes in the *T*_g_ in comparison to the sample obtained with the use of DCP. When ZnO was applied to crosslink XNBR, the glass temperatures were several degrees higher. The results confirmed that different concentrations of ZnO on the surface of the SiO_2_ particles had little effect on the glass temperature of the vulcanizates; which ranged from −19.7 °C for X1mS, −18.7 °C for X8mS and −17.7 °C for X20mS. 

Prior research indicated that the differential heat capacity (Δ*c_p_*) is directly proportional to the amount of the polymer that undergoes this transition [32]. Thus, the filled vulcanizates demonstrate a lower Δ*c_p_* value, as compared to the unfilled sample. It is believed that some fraction of the elastomer is immobilized on the filler’s surface, which provides a different *T*_g_, or even does not undergo a glass transition similar to the filler phase.

The results reported in Table 4 indicate that the values of heat capacity of the investigated samples containing different amounts of ZnO on SiO_2_ surface increased slightly with the increase of ZnO loading. This could be the result of changing the amounts of confined polymer chains, and, in this way, changing the structural mobility of the matrix rubber within the temperature of the glass transition.

**Figure 1:**
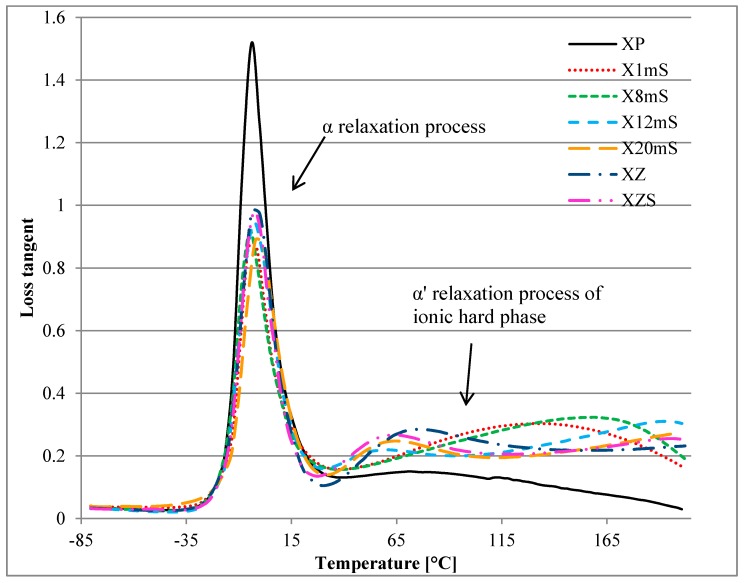
The effect of crosslinking substances on loss tangent (tan δ) of XNBR vulcanizates at 5 Hz versus temperature (*T*).

An assessment of the thermo-mechanical behavior of elastomers can be accomplished by examining the DMTA. In the lower temperature region, the α relaxation connected with segmental movements, the so called dynamic glass transition, can be observed. The vulcanizates investigated in this study varied in the type of crosslinking substance and concentration of ZnO on the surface of the SiO_2_ particles. However, we did not observe any significant changes in the glass transition temperature of the investigated vulcanizates (*T*_α (tan δ)_), taken at the maximum value of the tangent delta or loss modulus (*T*_α (*E″*)_) for 5 Hz, and these all varied within the margin of error, see *T*_α_ values in Table 4. Indeed the crosslinking density of the elastomers is known to be rather low in comparison with, e.g., phenol-formaldehyde crosslinked resins. Such low crosslinking density did not affect the segmental mobility of the elastomers chains. The DMTA traces of tan δ against temperature, at frequency 5 Hz, of the vulcanizates filled and unfilled, are shown in Figure 1. 

The results clearly reveal a very intense α transition dominating the spectra at around −5 °C, which must be related to the movement of soft polymer segments. The extracted dynamic glass transition values are listed in Table 4. Incorporation of ZnO containing curing substances into rubber compounds instead of DCP led to generating vulcanizates with slightly higher *T*_α_ values, except for the sample having 8ZnO/SiO_2_ (namely sample X8mS). The increasing values of *T*_α__(tan δ)_ suggest that the mobility of XNBR segments adsorbed on fillers surface is more severely confined in the three-dimensional filler network within the elastomer. The intensity of the relaxation transition of the soft segments (height of the tan δ peak) depends on the different crosslinking substances that are applied into the rubber compounds, see Table 4 and Figure 1. It is also interesting to note that there are slight differences in the tan δ peak height of filled XNBR vulcanizates, Table 4. In the case of unfilled sample the intensity of the relaxation transition of the soft segments was more pronounced. A decrease in the free volume did not occur, and this did not result in any restriction of the segmented macromolecules motions. Otherwise, when samples are filled with from 6 to 36 phr of fillers this rigid phase limits segmental mobility of polymer. 

*T*_g_ from the DMTA analysis are much lower as compared to those from the DSC measurements what results from the fact that the DSC is a static method of measurement as opposed to the DMTA where the sample is subjected to oscillating deformation.

**Figure 2:**
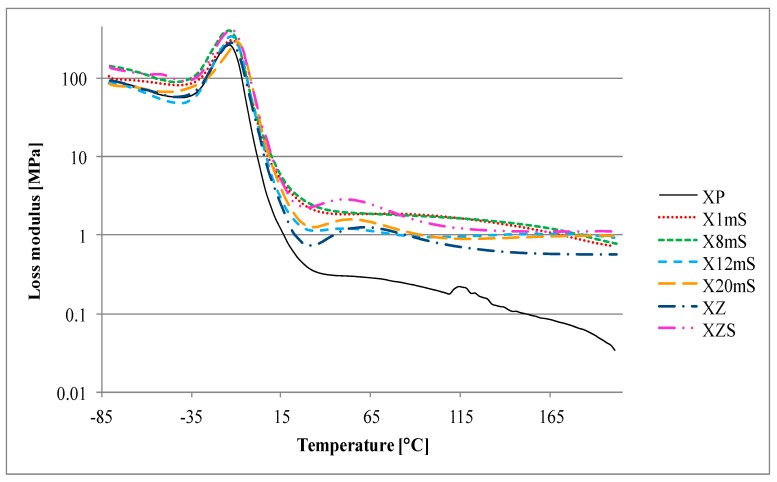
The temperature (*T*) dependency of loss modulus (*E″*) at 5 Hz for XNBR vulcanizates.

A second maximum (α′ relaxation) was detected in a wide range of temperature, above +50 °C. This can be assigned to the relaxation transition of the ionic hard phase arising from formation of ionic clusters or associates between zinc ions and functional –COOH groups present in the rubber [33]. Due to this interaction the mobility of the polymer macromolecules in the vicinity of the ionic region was restricted. These specific interactions have a labile character that can be proved, e.g., by stress relaxation measurements [26]. According to the labile nature of the ionic crosslinks, it is possible to detect the changes in tan δ connected with the relaxation process. This transition generates a maximum in the tan δ, denominated *T*_α′_*,* see Figure 1 and Figure 2. Significant differences are found in the case of filled vulcanizates regarding this parameter. This peak broadens and shifts to higher temperature for samples 1ZnO/SiO_2_ and 8ZnO/SiO_2_, which indicates that the fillers play a role in the ionic associations, and thus act as compatibilizers between the polymeric and ionic phases. It seems to be an optimum quantity of filler creating ionic links, under that is to say content of carboxyl groups, over which, if we introduce more filler, the ionic network is “plasticized”. 

This phenomenon caused an approach of *T*_α_ and *T*_α′_ peaks, which had been confirmed by the calculated values for *T*_α′_ − *T*_α_ listed in Table 4. The low intensity of the mentioned transition results from the fact that the mobility of the ionic phase was not very different below and above the transition temperature. 

**Figure 3:**
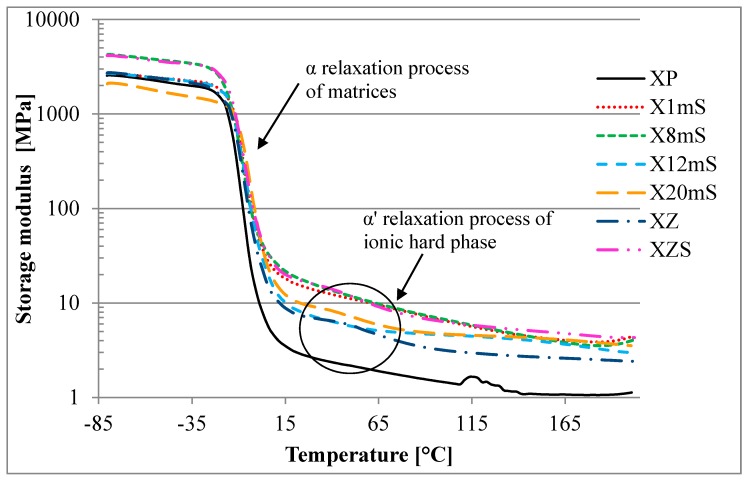
The temperature (*T*) dependency of storage modulus (*E′*) at 5 Hz for XNBR vulcanizates.

Figure 3 depicts the representative plots of the effect of temperature on dynamic elastic modulus (*E′*) of XNBR vulcanizates at frequency 5 Hz. 

The characteristic variation of this factor is observed for all of the samples. It is clear that *E′* of the samples discussed decreases rapidly with temperature as the composites pass from the glassy to the rubbery state, and tends to decrease very slowly when the temperature is increased from about +25 °C to 180 °C. Since it is possible to establish the temperature range when the storage modulus is more or less constant, up to 180 °C, it can be said that the ionic associations persist above *T*_α_. This behavior verifies that the ionic interactions induce some degree of crosslinking. 

Table 4 compares the storage modulus of the investigated vulcanizates between −75 °C and +20 °C (*E′_-75_* and *E′_20_*). The observed result is compatible with the fact that in the glassy state *E′* is always higher for industrial rubbers than for unfilled ones because of the mechanical reinforcement effect of the filler. The highest *E′_-75_* was reached when 8ZnO/SiO_2_ was used to cross-link XNBR. But increasing wt % of ZnO on silica’s surface to 20% resulted in obtaining a vulcanizate characterized by the lowest *E′_-75_*. The *E′_20_* follows a trend that is similar to *E′_-75_*. It is noteworthy that *E′_20_* displays a considerable increase when vulcanizates are filled by inorganic particles and had a higher degree of crosslinking. Vulcanizates containing silica and zinc oxide modified silica had a higher *E′* value, in the temperature range above zero degrees, probably due to the restricted mobility of the macromolecules chains that originated from higher interfacial bonding between functional groups on the surface of the silica and the XNBR rubber. The modulus in the rubbery region of the curves (*E′_20_*) is higher when modified silica particles were used, presumably due to the ionic interactions between the polymer and the filler. It is also evident that ZnO/SiO_2_ used as crosslinker affected the modulus of the vulcanizates. 

**Figure 4:**
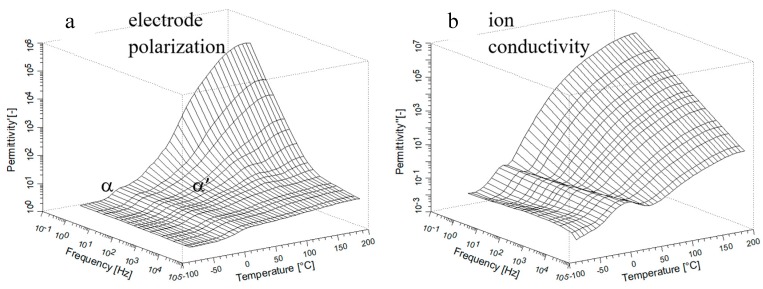
Frequency and temperature dependence of: (**a**) the real (permittivity′) and (**b**) imaginary (permittivity″) permittivity for vulcanizate crosslinked with ZnO/SiO_2_ (sample called X20mS).

The dielectric behavior of XNBR materials was also investigated to elucidate the effect of different crosslinking substances on polymer properties. Figure 4 shows the three-dimensional (3D) diagram of *ε″* as well as *ε′* for exemplary vulcanizates and their dependence on temperature and frequency. The *ε″* diagram clearly shows that, in addition to one relaxation in the low temperature region, a sharp increase of the dielectric loss at low frequencies and higher temperatures, which is correlated with ion-migration, which masks all of the relaxation processes in this region. However, the *ε′* dependency clearly indicates that in this region an additional relaxation process occurs.

**Figure 5:**
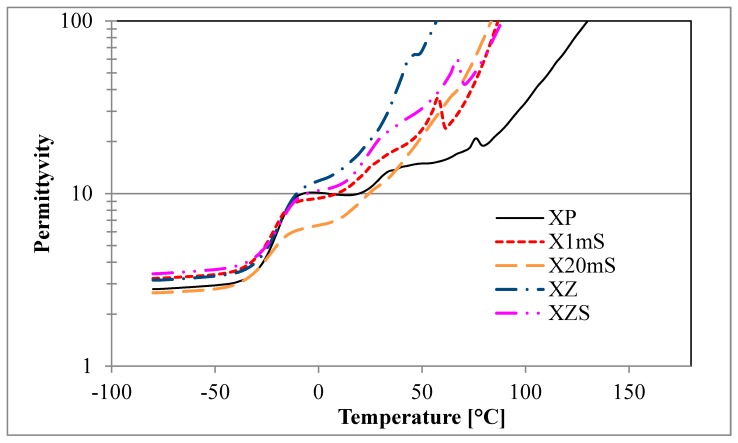
The temperature (*T*) dependency of permittivity (*ε′*) at 5 Hz for XNBR vulcanizates.

Figure 5 shows permittivity values (*ε′*) versus temperature at 5 kHz for all of the investigated XNBR vulcanizates. These results provide evidence of the presence of two relaxation processes for the discussed elastomers. However their positions and intensities are not very dependent on the different crosslinking substance used to prepare the vulcanizate. 

**Figure 6:**
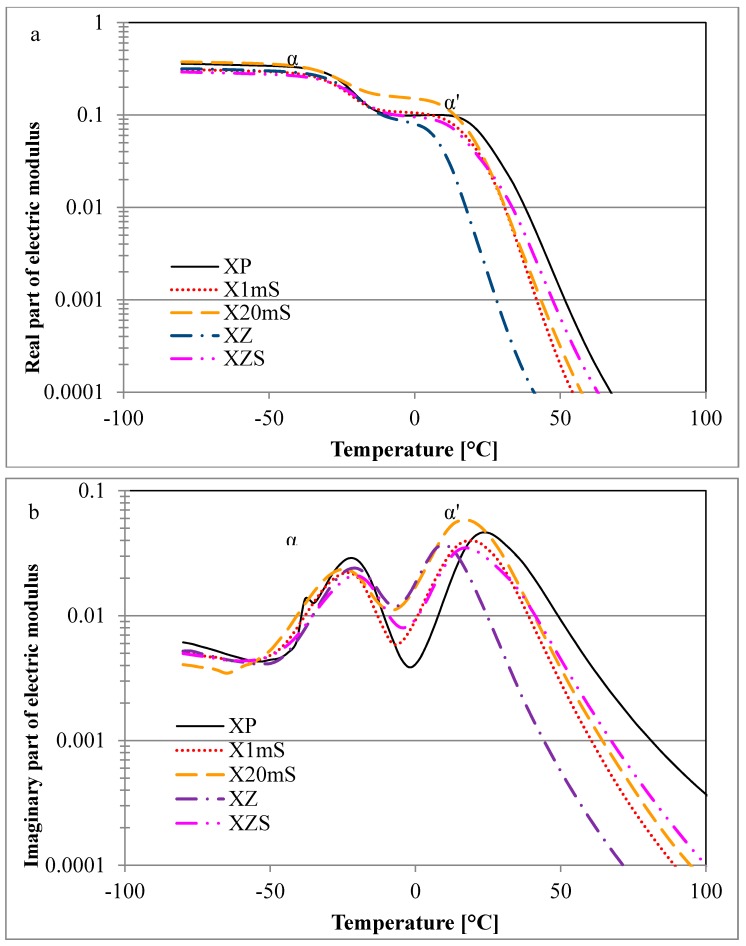
(**a**) The real (*M′*) and (**b**) the imaginary (*M″*) part of electric modulus versus temperature (*T*) at 5 Hz for studied XNBR vulcanizates.

In the low temperature region, there is an α relaxation of XNBR due to the segmented motions of the polymer chains. The presence of an *α*′ relaxation, noted at higher temperatures on the curves, could be assigned to the ionic hard phase arising from interactions between the carboxyl groups and zinc ions, similar to DMTA results. This provides further evidence of the existence of specific interactions between carboxyl groups on the rubber and zinc oxide incorporated on the surface of the silica particles, or directly introduced into the rubber compound during its preparation. Thus, non-conventional elastomers’ crosslinks were formed, which are discussed above, and are able to move under applied field. When the temperature is decreased at low frequencies, the ability of the ions to migrate is restricted. The huge increase in the value of *ε′* at low frequencies and high temperatures is correlated with electrode polarization. DRS is an easy way to detect the relaxation processes at high temperatures, even if the ionic conductivity dominates, by analysis of the electric modulus, the real *M′* and imaginary *M″* part [31]. Figure 6 shows that two well defined *α* and α′ relaxations were observed when *M′* and *M″* are plotted as a function of temperature. 

**Figure 7:**
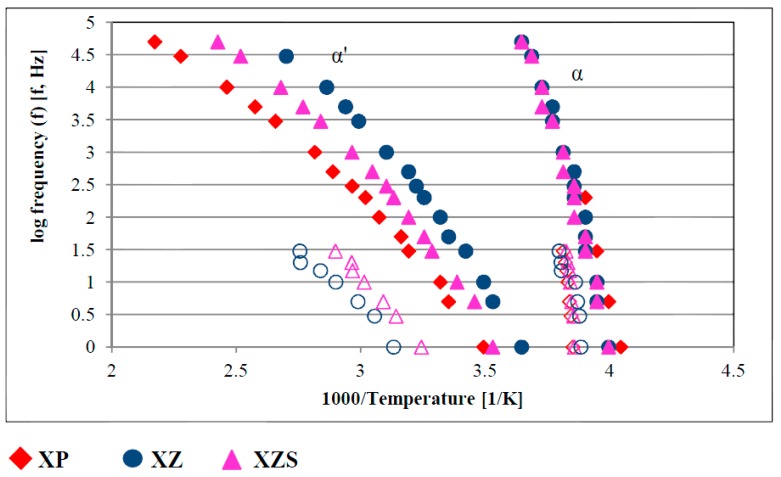
Activation diagrams of XNBR vulcanizates containing different crosslinking agents (full points—DRS and open points—DMA).

**Figure 8:**
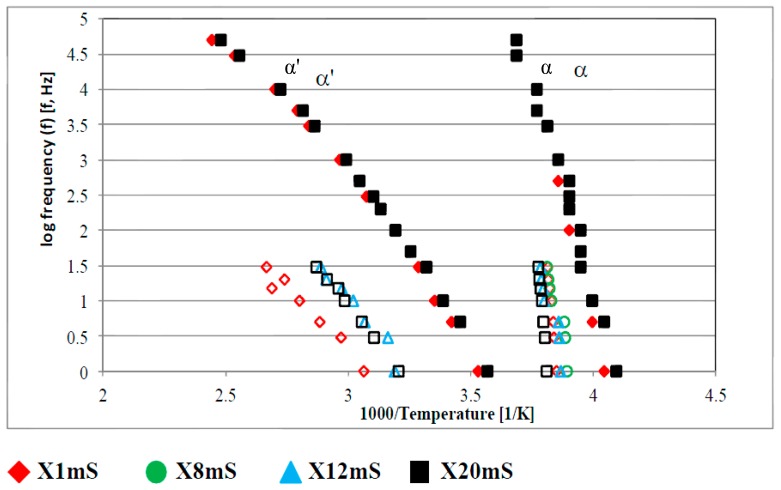
Activation diagrams of XNBR vulcanizates containing silica modified with different amount of zinc oxide (ZnO/SiO_2_) (full points—DRS and open points—DMA).

It is interesting to compare both dielectric and mechanical results as this provides some insight into the molecular processes due to sensitivity of fluctuations in dipole moments (DRS) and internal stresses (DMTA). The activation diagrams of the relaxation processes are depicted in Figure 7 and Figure 8 as activation plots. 

By comparing the dielectric and mechanical results, it can be seen that the α and α′ peaks in the dielectric experiments occur at a slightly lower temperature in comparison to the values that are observed from the mechanical experiments, but this can be caused by the fact that the points in DRS are taken from *M″*(*T*) curves, which are shifted in comparison to standard permittivity dependencies. Nevertheless, one can see that the curves representing DRS (full points) and DMTA (open points) results have similar slopes, which indicate that the relaxations that are seen in both techniques have the same origin, i.e., α relaxation comes from segmental motions, so called dynamic glass transition, and the α′ relaxation is connected with movements of the ionic hard phase or segments directly connected to such crosslinks.

## 4. Conclusions

In this study, the properties of carboxylated butadiene-acrylonitrile rubber (XNBR) vulcanizates, and the effect of both a new crosslinking substance and new filler with a core-shell structure, were investigated. Zinc oxide was incorporated onto the surface of silica particles (ZnO/SiO_2_), thereby creating ionic crosslinks within XNBR matrix. The formation of ionic crosslinks within the vulcanizates influence the molecular dynamics of the investigated polymer materials.

Elastomer-silica composite materials and unfilled samples were investigated by a series of thermoanalytical measurements, e.g., DRS, DSC, DMTA, and a supplementary measurement: equilibrium swelling. DSC measurements performed on the particle filled samples showed that introducing ZnO/SiO_2_ as a crosslinking substance into the carboxylate functionalized rubber compound led to no significant changes in the glass temperatures (*T_g_*) in comparison to the *T_g_* of the sample crosslinked with DCP. It appeared that the heat capacity values of the investigated samples containing different amounts of ZnO on the surface of SiO_2_ increased slightly, with an increase of ZnO loading. On the basis of DMTA, the vulcanizates investigated in this study vary with the kind of crosslinking substance and concentration of ZnO on SiO_2_ particle surface, this affects the value that is obtained for *T*_α_, which is taken as the maximum value of tan δ under measurement at 5 Hz. The change in *T*_α_ can be indicative of the constrained effect on the segmental mobility. In the case of unfilled samples, the intensity of the relaxation transition of the soft segments was more pronounced due to no decrease of free volume or any restriction of the motion of the segmented macromolecules occurred. Vulcanizates containing fillers had higher *E′* values in the temperature range above zero degrees. Due to the interactions between the polymer and the filler, the modulus in the rubbery region of the curves (*E′_20_*) is higher when ZnO/SiO_2_ was used as fillers. It is also evident that ZnO/SiO_2_ acts as crosslinker and affects the modulus values of the vulcanizates. A second maximum, α′ relaxation, characterizing the relaxation transition of the ionic hard phase arising due to formation of ionic clusters or associates between zinc ions and functional –COOH groups on the backbone of the rubber was detected in a wide range of temperature, above +50 °C. Moreover, this peak broadens and shifts to higher temperature, which can testify that the fillers interact via ionic associations, and thus act as compatibilizers between the polymeric and ionic phases. Since it was possible to establish the temperature range when the storage modulus is more or less constant, up to 180 °C, it can be said the ionic associations persist above *T*_α_. This behavior confirms that ionic aggregations induce some degree of crosslinking.

The dielectric measurements on the XNBR materials clearly indicated the presence of two relaxation processes (α and α′ similar to the DMTA results) for the discussed elastomers, however, their positions and intensities do not depend on the crosslinking substance that is used to prepare the vulcanizate. 

## Figures and Tables

**Table 1:** Description of the materials used in the study.

Materials	Properties	Suppliers
Carboxylated butadiene-acrylonitrile rubber XNBR (trade name Krynac 7,5X)	6.4% by weight of carboxylic groups; 26.3% by weight of acrylonitrile mers; density = 0.99 g/cm^3^	Lanxess, Warsaw, Poland
Precipitated silica SiO_2_ (trade name Zeosil 175MP)	Surface area = 143 m^2^/g [26]	Solvay, Brussels, Belgium
Dicumyl peroxide (DCP)	Melting point = 39–41 °C	Fluka, Poznan, Poland
Zinc oxide (ZnO)	Density = 5.6 g/cm^3^	Huta Bedzin, Bedzin, Poland
Modified silica particles (ZnO/SiO_2_)	Concentration of ZnO on the surface 1%–20%	Synthesized by the process discussed in reference [27]

**Table 2:** Formulation of the rubber blends.

	Symbol of Sample	XP	XZ	XZS	X1mS	X8mS	X12mS	X20mS
Compounds								
XNBR		100	100	100	100	100	100	100
DCP		2	-	-	-	-	-	-
ZnO		-	6	6	-	-	-	-
SiO_2_		-	-	30	-	-	-	-
1ZnO/SiO_2_		-	-	-	30	-	-	-
8ZnO/SiO_2_		-	-	-	-	30	-	-
12ZnO/SiO_2_		-	-	-	-	-	30	-
20ZnO/SiO_2_		-	-	-	-	-	-	30

**Table 3:** The crosslink density of carboxylated butadiene-acrylonitrile rubber (XNBR) vulcanizates.

Symbol of Sample	ν	*Δ*ν_A_*/*ν (×)100%
XP	7.70	2.30
XZ	3.91	10.4
XZS	5.11	24.7
X1mS	3.55	29.9
X8mS	4.08	38.0
X12mS	5.68	39.3
X20mS	7.21	46.1

ν—crosslink density, (×)10^−5^ (mole/cm^3^); *Δ*ν_A_—decrease of crosslink density under the influence of ethylenediamine.

**Table 4:** The results of thermal analysis of XNBR vulcanizates.

Compound	*T*_g (DSC)_ (°C)	Δ*c_p_* (Jg^−1^K^−1^)	*T*_α (*E″*)_ (°C)	*T*_α (tan δ)_ (°C)	*T*_α′ (tan δ)_ (°C)	*T*_α′_ − *T*_α_ (°C)	Height of tan δ at *T_α_*	Height of tan δ at *T*_α′_	*E′_-75_* (MPa)	*E′_20_* (MPa)
XP	−19.2	0.25	−12.7	−4.1	-	-	1.52	-	2529.5	3.1
XZ	−14.5	0.08	−14.7	−3.9	76.0	79.9	0.98	0.28	2710.2	7.7
XZS	−15.8	0.14	−13.3	−2.7	63.0	65.8	0.98	0.27	4103.6	17.7
X1mS	−19.7	0.01	−12.6	−1.8	129.6	131.4	0.87	0.30	2687.2	16.4
X8mS	−18.7	0.09	−15.6	−4.8	158.9	163.7	0.92	0.32	4199.6	19.5
X12mS	-	-	−14.1	−3.0	59.8	62.9	0.94	0.22	2655.1	8.5
X20mS	−17.7	0.11	−13.3	−2.2	63.9	66.1	0.89	0.25	2109.1	10.7

*T*_g_—glass transition temperature determined by differential thermal calorimetry (DSC); Δ*c_p_*—heat capacity; *T*_α (*E″*)_, *T*_α (tan δ)_—temperature respectively of *E″*(*T*) or mechanical tan δ (*T*) maxima, connected to α relaxation process at a frequency 5 Hz; *T*_α′ (tan δ)_—temperature of mechanical tan δ (*T*) maximum, connected to ionic crosslinks relaxation, at a frequency 5 Hz; height of tan δ at *T*_α_ *or T*_α′_—mechanical tangent delta related to *T*_α_ or *T*_α′_ at a frequency 5 Hz; *E′_20_*, *E′_-75_*—storage modulus of vulcanizates at a frequency 5 Hz, respectively, at −75 °C and 20 °C.

## References

[1] López-Manchado M.A., Valentin J.L., Carretero J., Barroso F., Arroyo M. (2007). Rubber network in elastomer nanocomposites. Eur. Polym. J..

[2] Ohbi D.S., Purewal T.S., Shah T., Siores E. (2008). Crosslinking reaction mechanism of diisopropyl xanthogen polysulfide accelerator in bromobutyl elastomer for medical device applications. J. Appl. Polym. Sci..

[3] Alam N., Mandal S.K., Debnath S.C. (2012). Bis(N-benzyl-piperazine)thiuram disulfide and dibenzothiazyl disulfide as synergistic safe accelerators in the vulcanization of natural rubber. J. Appl. Polym. Sci..

[4] Przybyszewska M., Zaborski M., Jakubowski B., Zawadiak J. (2009). Zinc chelates as new activators for sulphur vulcanization of acrylonitrile-butadiene elastomers. Express Polym. Lett..

[5] Naskar K., Noordermeer J.W.M. (2004). Multifunctional peroxides as a means to improve properties of dynamically vulcanized PP/EPDM blends. Kautsch. Gummi Kunstst..

[6] Owczarek M., Zaborski M. (2005). Chlorosulfonated polyethylene cross-linked with aminosilanes. Kautsch. Gummi Kunstst..

[7] Bhowmick A.K., Stephens H.L. (2001). Handbook of Elastomers.

[8] Ibarra L., Alzorriz M. (2007). Ionic elastomers based on carboxylated nitrile rubber and magnesium oxide. J. Appl. Polym. Sci..

[9] Ibarra L., Rodrigues A., Mora I. (2007). Ionic nanocomposites based on XNBR-OMg filled with layered nanoclays. Eur. Polym. J..

[10] Ibarra L., Rodrigues A., Mora-Barrantes I. (2009). Crosslinking of carboxylated nitrile rubber (XNBR) induced by coordination with anhydrous copper sulfate. Polym. Int..

[11] Ibarra L., Rodrigues A., Mora-Barrantes I. (2008). Crosslinking of unfilled carboxylated nitrile rubber with different systems: Influence on properties. J. Appl. Polym. Sci..

[12] Tobolski A.V., Lyons P.F., Hata N. (1968). Ionic clusters in high-strength carboxylic rubbers. Macromolecules.

[13] Eisenberg A. (1970). Clustering of ions in organic polymers. A theoretical approach. Macromolecules.

[14] Mora-Barantes I., Malmierca M.A., Valentin J.L., Rodriguez A., Ibarra L. (2012). Effect of covalent cross-links on the network structure of thermo-reversible ionic elastomers. Soft Matter..

[15] De Luca M.A., Jacobi M.M., Orlandini L.F. (2009). Synthesis and characterization of elastomeric composites prepared from epoxydized styrene butadiene rubber, 3-aminopropyltriethoxysilane and tetraethoxysilane. J. Sol-Gel Sci. Technol..

[16] Chokanandsombad Y., Sirisinha C. (2013). MgO and ZnO as reinforcing fillers in cured polychloroprene rubber. J. Appl. Polym. Sci..

[17] Przybyszewska M., Zaborski M. (2009). The effect of zinc oxide nanoparticle morphology on activity in crosslinking of carboxylated nitrile elastomers. Express Polym. Lett..

[18] Gaca M., Zaborski M. (2016). The properties of elastomers obtained with the use of carboxylated acrylonitrile-butadiene rubber and new crosslinking substances. Polimery.

[19] Pietrasik J., Gaca M., Zaborski M., Okrasa L., Boiteux G., Gain O. (2009). Studies of molecular dynamics of carboxylated acrylonitrile-butadiene rubber composites containing in situ synthesized silica particles. Eur. Polym. J..

[20] Czech P., Okrasa L., Ulanski J., Boiteux G., Mechin F., Cassagnau P. (2007). Studies of the molecular dynamics in polyurethane networks with hyperbranched crosslinkers of different coordination numbers. J. Appl. Polym. Sci..

[21] Owczarek M., Zaborski M., Paryjczak T., Boiteux G., Gain O. (2003). New type of inorganic filler with a core-shell structure. Macromol. Symp..

[22] Owczarek M., Zaborski M. (2004). Chlorosulfonated polyethylene elastomers containing zinc oxide incorporated on SiO_2_. Kautsch. Gummi Kunstst..

[23] Chen Y., Xu C. (2011). Crosslink network evolution of nature rubber/zinc dimethacrylate composite during peroxide vulcanization. Polym. Compos..

[24] Chen Y., Xu C. (2012). Stress-strain behaviors and crosslinked networks studies of natural rubber-zinc dimethacrylate composites. J. Macromol. Sci. Part B.

[25] Xu C., Chen Y., Zeng X. (2012). A study on the crosslink network evolution of magnesium dimethacrylate/natural rubber composite. J. Appl. Polym. Sci..

[26] Zaborski M., Owczarek M., Paryjczak T., Każmierczak A. (2002). Właściwości karboksylowanego kauczuku butadienowo-akrylonitrylowego usieciowanego za pomocą układu ZnO/SiO_2_. Polimery.

[27] Zaborski M., Paryjczak T., Kaźmierczak A., Albińska J. (2002). Charakterystyka fizykochemicznych właściwości nieorganicznych składników mieszanin polimerowych o budowie jądro-powłoka. Polimery.

[28] Flory P.J. (1950). Statistical mechanics of swelling of network structures. J. Chem. Phys..

[29] Shinichi Y., Kenji T., Nobuaki N., Shoichi K., Hitoshi T., Eisaku H. (1992). Dielectric relaxation studies on water absorption of ethylene ionomers. Macromolecules.

[30] Vondracek P., Pouchaleon A. (1990). Ammonia-induced tensile set and swelling in silica-filled silicone rubber. Rubb. Chem. Technol..

[31] Kremer F., Schönhals A. (2003). Broadband Dielectric Spectroscopy.

[32] Vidal A., Haidar B. (2007). Filled elastomers: Characteristics and properties of interfaces and their role in reinforcement processes. Soft Mater..

[33] Gaca M., Zaborski M. (2016). The properties of ethylene–propylene elastomers obtained with the use of a new cross-linking substance. J. Term. Anal. Calorim..

